# A Human Monoclonal Antibody with Neutralizing Activity against Highly Divergent Influenza Subtypes

**DOI:** 10.1371/journal.pone.0028001

**Published:** 2011-12-05

**Authors:** Nicola Clementi, Donata De Marco, Nicasio Mancini, Laura Solforosi, Guisella J. Moreno, Larisa V. Gubareva, Vasiliy Mishin, Andrea Di Pietro, Elisa Vicenzi, Antonio G. Siccardi, Massimo Clementi, Roberto Burioni

**Affiliations:** 1 Laboratorio di Microbiologia e Virologia, Università Vita-Salute San Raffaele, Milano, Italia; 2 Virus Surveillance and Diagnosis Branch, Influenza Division, NCIRD, Centers for Disease Control and Prevention, Atlanta, Georgia, United States of America; 3 Viral Pathogens and Biosafety Unit, Divisione di Immunologia, Trapianti e Malattie infettive, Istituto Scientifico San Raffaele, Milano, Italia; 4 Istituto Scientifico San Raffaele, Milano, Italia; 5 Dipartimento di Biologia e Genetica per le Scienze Mediche, Università di Milano, Milano, Italia; Erasmus Medical Center, The Netherlands

## Abstract

The interest in broad-range anti-influenza A monoclonal antibodies (mAbs) has recently been strengthened by the identification of anti-hemagglutinin (HA) mAbs endowed with heterosubtypic neutralizing activity to be used in the design of “universal” prophylactic or therapeutic tools. However, the majority of the single mAbs described to date do not bind and neutralize viral isolates belonging to highly divergent subtypes clustering into the two different HA-based influenza phylogenetic groups: the group 1 including, among others, subtypes H1, H2, H5 and H9 and the group 2 including, among others, H3 subtype. Here, we describe a human mAb, named PN-SIA28, capable of binding and neutralizing all tested isolates belonging to phylogenetic group 1, including H1N1, H2N2, H5N1 and H9N2 subtypes and several isolates belonging to group 2, including H3N2 isolates from the first period of the 1968 pandemic. Therefore, PN-SIA28 is capable of neutralizing isolates belonging to subtypes responsible of all the reported pandemics, as well as other subtypes with pandemic potential. The region recognized by PN-SIA28 has been identified on the stem region of HA and includes residues highly conserved among the different influenza subtypes. A deep characterization of PN-SIA28 features may represent a useful help in the improvement of available anti-influenza therapeutic strategies and can provide new tools for the development of universal vaccinal strategies.

## Introduction

Influenza, one of the diseases that has shaped human history [1], [2], still has an evident clinical and socio-economical impact [3], [4]. The 2009 pandemic has raised several major concerns related to the few prophylactic and therapeutic measures available. Antiviral compounds have drawbacks caused by the rapid emergence of drug-resistant isolates [5], [6], require prompt administration to be effective [7], and have several associated side-effects especially in high-risk categories, including children and pregnant women [8], [9]. Additionally, the vaccinal strategy is exposed to the annual risk of being ineffective due to possible mismatches between the predicted strains included in the vaccine and those actually in circulation; moreover, it would not engender a prompt response in pandemic settings [10]. In this scenario, new broad–range “universal” anti-influenza strategies are required [11], [12]. In particular, it would be important to identify and eventually elicit what has recently been described as an unusually extreme broad-range immunity directed against broadly conserved viral regions, differing from the more common and restricted immunity directed against highly variable regions [12]. A number of approaches have already been proposed in literature [10], [12], [13], [14], [15], [16], but a pivotal role, both in the prophylactic and therapeutic field, may be played by the availability of broad-range neutralizing human monoclonal antibodies (mAbs) allowing the identification of human B epitopes widely shared among different influenza subtypes [11], [12]. Indeed, it is accepted that antibodies are key players in natural protection against influenza viruses, and that hemagglutinin (HA) is the main target for the virus-neutralizing antibody response [17]. However, although a single influenza infection provides lifelong immunity against the infecting virus and a limited number of antigenically correlated strains, the host can remain susceptible to infection with an antigenically drifted variant due to HA variability [18].

HA is the major glycoprotein of the influenza virus; it binds sialic acid on the surface of the cells through its globular head (HA1 domain) and makes possible the fusion of the viral envelope with the endosomal membranes through its stalk region (mainly formed by the HA2 domain) [17]. The sixteen known subtypes of HA, sharing between 40% and 60% amino acid sequence identity, have been clustered in two distinct phylogenetic groups: group 1 (H1, H2, H5, H6, H8, H9, H11, H12, H13, and H16) and group 2 (H3, H4, H7, H10, H14, and H15) [12], [14]. The subtypes, H1, H2, H5 and H9 in group 1, and H3 and H7 in group 2 have been isolated in humans and in particular H1, H2 and H3 subtypes have been responsible of the reported influenza pandemic outbreaks. In this study, we describe a human mAb, named PN-SIA28, that is capable of neutralizing all tested group 1 isolates, as well as isolates belonging to H3N2, the only group 2 subtype capable, so far, of causing a pandemic.

## Results

### Neutralizing activity of PN-SIA28

The binding and neutralizing features of PN-SIA28 were initially studied using the Fab fragment molecule produced in *E. coli*, demonstrating that Fab PN-SIA28 recognizes an epitope on the stem region of HA and is able to strongly neutralize all tested H1N1 strains [19], [20].

**Figure 1:**
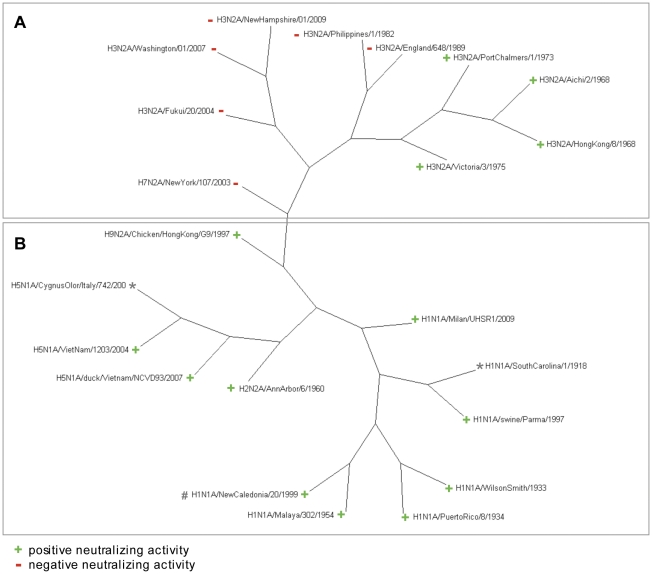
PN-SIA28 Neutralizing activity. Influenza hemagglutinin unrooted phylogenetic tree of the different viral strains tested in neutralization assays with PN-SIA28. Viral isolates belonging to group 1 and group 2 are presented in the box B and box A, respectively. A green ‘+’ indicates positive neutralizing activity, a red ‘−’ indicates negative neutralizing activity. As reported in the text, PN-SIA28 is able to neutralize all of the group 1 strains and is also able to neutralize all of the H3N2 isolates spanning 1968 and 1975. *Recombinant HA from (H1N1) A/South Carolina/1/1918 pandemic strain was previously shown to be bound by PN-SIA28 [19], [20]. Analogously, recombinant HA from H5N1 A/Cygnus Olor/Italy/742/2005 was recognized by PN-SIA28 (data not shown). # H1N1 A/New Caledonia/20/1999 was previously shown to be neutralized by PN-SIA28 as Fab fragment [19], [20].

It is well documented in the literature that bivalency of a whole IgG molecule may be an essential features for the biological activity of a mAb [21], [22], [23], [24]. For this reason, in this work, to evaluate PN-SIA28 features as whole IgG molecule, IgG PN-SIA28 was generated and tested in different neutralization assays against human, swine and avian influenza A viruses belonging to both HA based phylogenetic groups and encompassing all subtypes responsible of described pandemic events. The results obtained showed that IgG PN-SIA28 strongly neutralizes viruses belonging to the group 1 as well as those of the group 2. More in details, IgG PN-SIA28 neutralized all the H1N1 tested viruses with an half maximal inhibitory concentration (IC_50_) ranging between 0.4–3.7 µg/ml, the H5N1 viruses with IC_50_ ranging between 0.9–2.8 µg/ml, the H2N2 subtype isolate with an IC_50_ of 0.8 µg/ml and the H9N2 subtype strain with an IC_50_ of 0.9 µg/ml (Table 1, Figure S1). More interestingly, IgG PN-SIA28, also potently neutralizes H3N2 viruses circulating in the years from 1968 to 1975 with IC_50_ ranging between 0.8–2.6 µg/ml (Table 1, Figure S2) despite the great phylogenetic distance of these viruses compared to those belonging to group 1 (Figure 1). No detectable neutralizing activity was observed against more recent H3N2 tested viruses (Table 1, Figure 1 and S3) as well as for the H7N2 tested isolate.

**Table 1:** PN-SIA28 neutralizing activity against all tested isolates.

Subtype	Strain	IC_50_ a µg/ml (95% CI^b^)
H1N1	A/Wilson Smith/33	0.4 (0.3–0.5)
	A/Puerto Rico/8/34	0.8 (0.2–2.9)
	A/Malaya/302/54	0.7 (0.3–1.4)
	A/Milan/UHSR1/2009	1.5 (1.3–1.6)
	A/swine/Parma/1/97	3.7 (2.8–4.9)
H2N2	A/Ann Arbor/6/60	0.8 (0.1–4.8)
H5N1	A/Vietnam/1203/2004 clade 1	0.9 (0.1–8.1)
	A/duck/Vietnam/NCVD98/2007 clade 2.3.4	2.8 (1.3–5.9)
H9N2	LAIV A/chicken/Hong Kong/G9/97	0.9 (0.2–3.4)
H3N2	A/Aichi/2/68	1.3 (0.7–2.5)
	A/Hong Kong/8/68	0.8 (0.5–1.4)
	A/Port Chalmers/1/73	2.6 (1.8–3.7)
	A/Victoria/3/75	1 (0.4–2.3)
	A/Philippines/01/82	>10
	A/England/648/89	>10
	A/Fukui/20/2004	>10
	A/Washington/01/2007	>30
	A/New Hampshire/01/2009	>30
H7N2	A/New York/107/2003	>10

a IC_50_: Half maximal inhibitory concentration expressed in µg/ml.

b CI: confidence interval.

### Epitope mapping

#### Screening of a random 12-mer peptide phage displayed library

**Figure 2:**
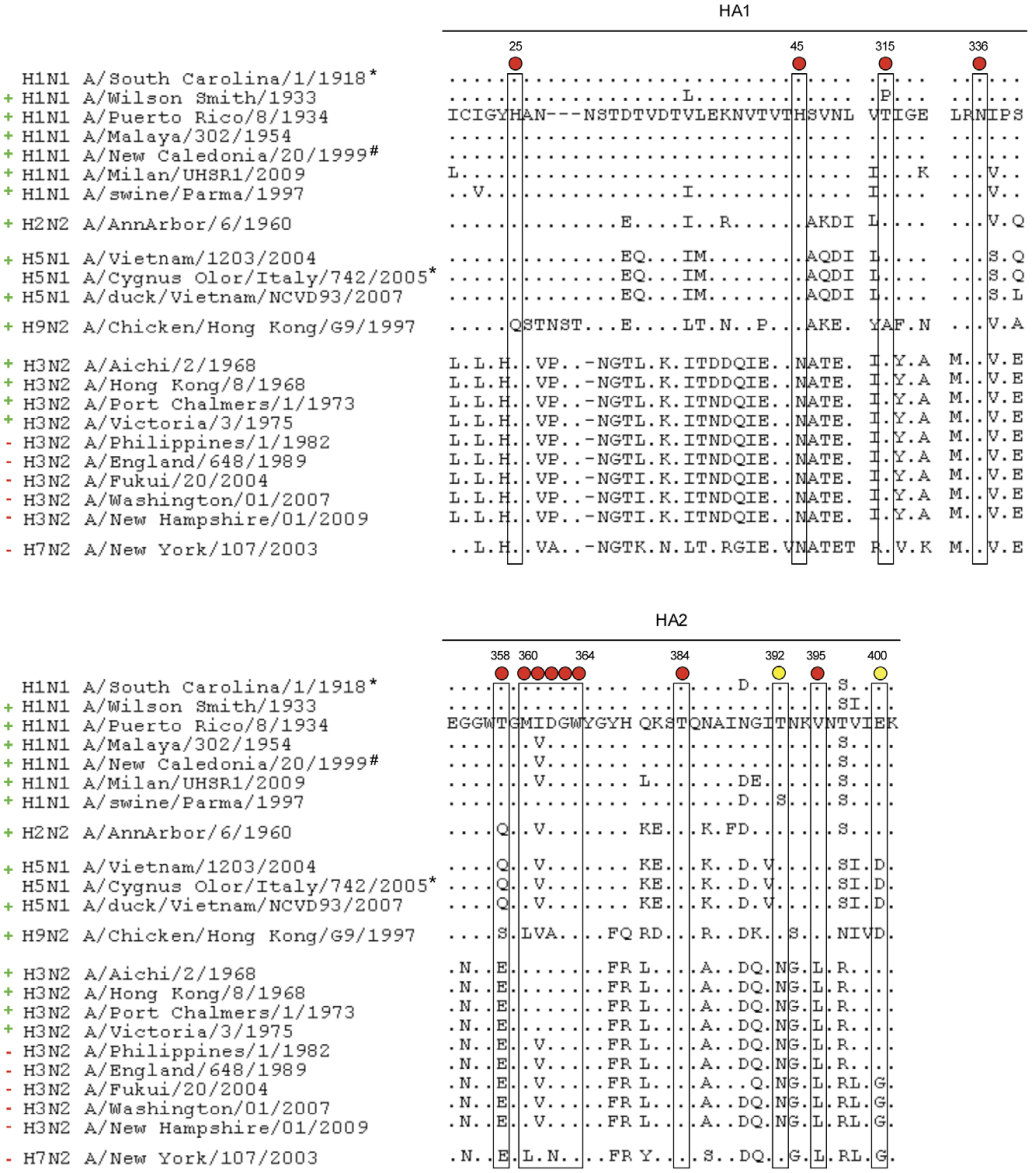
Sequence conservation in hemagglutinin groups and subtypes. Boxes indicate mutated residues which decrease PN-SIA28 binding to mutated HAs. Circles on the top indicate PN-SIA28 percent binding to each HA alanine mutants compared to binding to wild-type HA: red 25% binding, yellow 50–75% binding. Sequence numbering is based on H1N1 A/PR/8/34 coding region (GenBank accession number ABO21709). Neutralizing activity of PN-SIA28 against each strain is highlighted by a green ‘+’ or a red ‘−’ on the left, indicating neutralizing activity and no neutralizing activity, respectively. *Recombinant HA from H1N1 A/South Carolina/1/1918 pandemic strain was previously shown to be bound by PN-SIA28 [19], [20]; analogously, recombinant HA from H5N1 A/Cygnus Olor/Italy/742/2005 was recognized by PN-SIA28 (data not shown). # H1N1 A/New Caledonia/20/1999 was previously shown to be neutralized by PN-SIA28 as Fab fragment [19], [20].

In order to select peptides able to bind antibody PN-SIA28 that could possibly indicate the region that the antibody recognizes on the HA, a phagemidic random 12-mer peptide library was screened. After three rounds of panning, PN-SIA28 binding phages were eluted and used to infect *E. coli* XL1-Blue for an ELISA screening on PN-SIA28. Twenty-five positive clones were sequenced. An *in silico* analysis was performed, using Mimox and Pepitope servers, comparing the selected peptides to the available crystal structure of H1N1 A/South Carolina/1918 (A/SC/1918) (ID code 1RD8) and A/Puerto Rico/8/34 (A/PR/8/34) hemagglutinins (ID code 1RU7). This study allowed the identification of several possible residues located on the stem region of HA potentially involved in the binding of PN-SIA28: Asn336, Ile337, Pro338, Trp357 and Thr358 (sequence numbering refers to A/PR/8/34, GenBank accession number ABO21709) (Figure 2).

#### In vitro selection of escape mutants

To further define the region bound by PN-SIA28, an assay aiming at the evaluation of PN-SIA28 capability to induce escape mutant was performed. A/PR/8/34 H1N1 virus was cultured under constant selective pressure of PN-SIA28, and after several rounds of cell infections, two escape mutants were generated. Sequencing analysis of the generated neutralization escape variants revealed two different mutants carrying each a single amino acidic mutation in position 361 (Ile361Thr) and in position 362 (Asp362Gly) in the HA2 subunit compared to wild type.

#### Alanine scanning mutagenesis study

On the basis of these results, HA mutants carrying an alanine substitution in position 361 (Ile361Ala) or in position 362 (Asp362Ala) were generated and the PN-SIA28 binding to the mutants was evaluated by FACS analysis evidencing that PN-SIA28 was not longer able to bind to the mutated HAs. In order to identify other amino acidic residues involved in the binding of PN-SIA28 to HA, a large panel of HA-alanine mutants was generated (Table S1). FACS analysis showed that binding of PN-SIA28 to HA was decreased by His25Ala, His45Ala mutants on HA1 and Thr358Ala, Met360Ala, Ile361Ala, Asp362Ala, Gly363Ala, Trp364Ala, Thr384Ala and Val395Ala mutants on HA2. (Figure 2). All other mutations listed in the Table S1 did not have any effect on PN-SIA28 binding. An extra HA-mutant in position 361 (Ile361Val) was generated. Indeed, in position 361 either an Isoleucine or a Valine residue can be present in different isolates belonging to either to group 1 either to group 2 (Figure 2). No binding reduction of PN-SIA28 to this mutant was observed.

**Figure 3:**
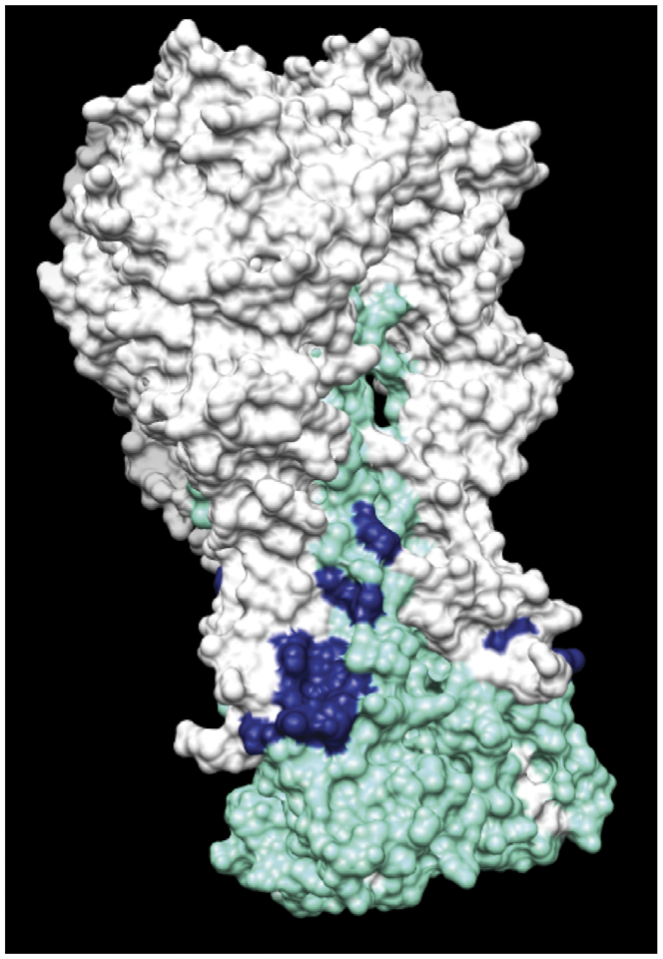
Position of the residues on the surface of (H1N1) A/PR/8/34 influenza hemagglutinin trimer decreasing the binding of PN-SIA28. White areas indicate the HA1 domains of the HA trimer, whereas cyan areas indicate the HA2 domains. Blue regions depict the mutated residues inhibiting PN-SIA28 binding to HA according to Figure 2.

Based on the results obtained with the HA mutants, an *in silico* analysis on the HA crystal structure of A/SC/1918 and A/PR/8/34 was carried out for the amino acid residues that the alanine scanning study showed to influence the binding of PN-SIA28 to HA. The analysis confirmed that the residues identified lie on the stem region of HA, that they belong to the HA1 and HA2 subunits and that they are exposed on the surface of the HA molecule (Figure 3).

**Figure 4:**
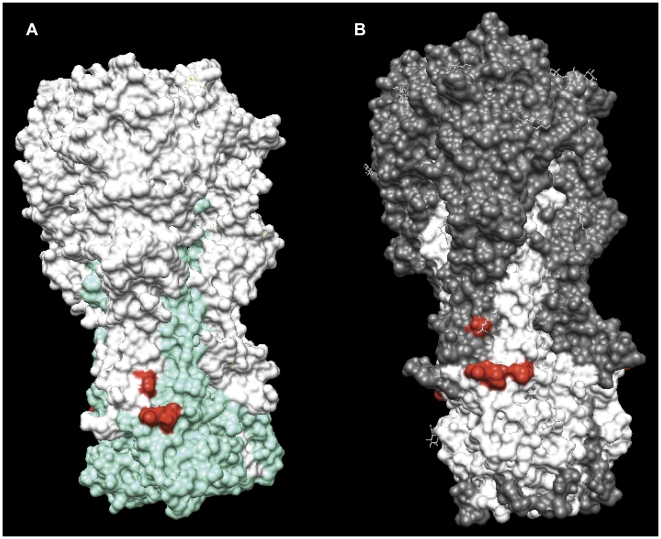
Comparison of the core of PN-SIA28 epitope on the surface of H1 and H3 trimers. (A) (H1N1) A/PR/8/34 HA: white areas indicate the HA1 domains of H1 trimer whereas cyan areas indicate its HA2 domains. (B) (H3N2) A/Aichi/2/68 hemagglutinin: grey areas indicate the HA1 domains of H3 trimer whereas white areas indicate its HA2 domains. On both pictures, red regions indicate the residues 25 and 45 on HA1 and residues 361 and 362 on HA2 that, when mutated, were able to inhibit PN-SIA28 binding both to H1 and H3 HAs.

**Figure 5:**
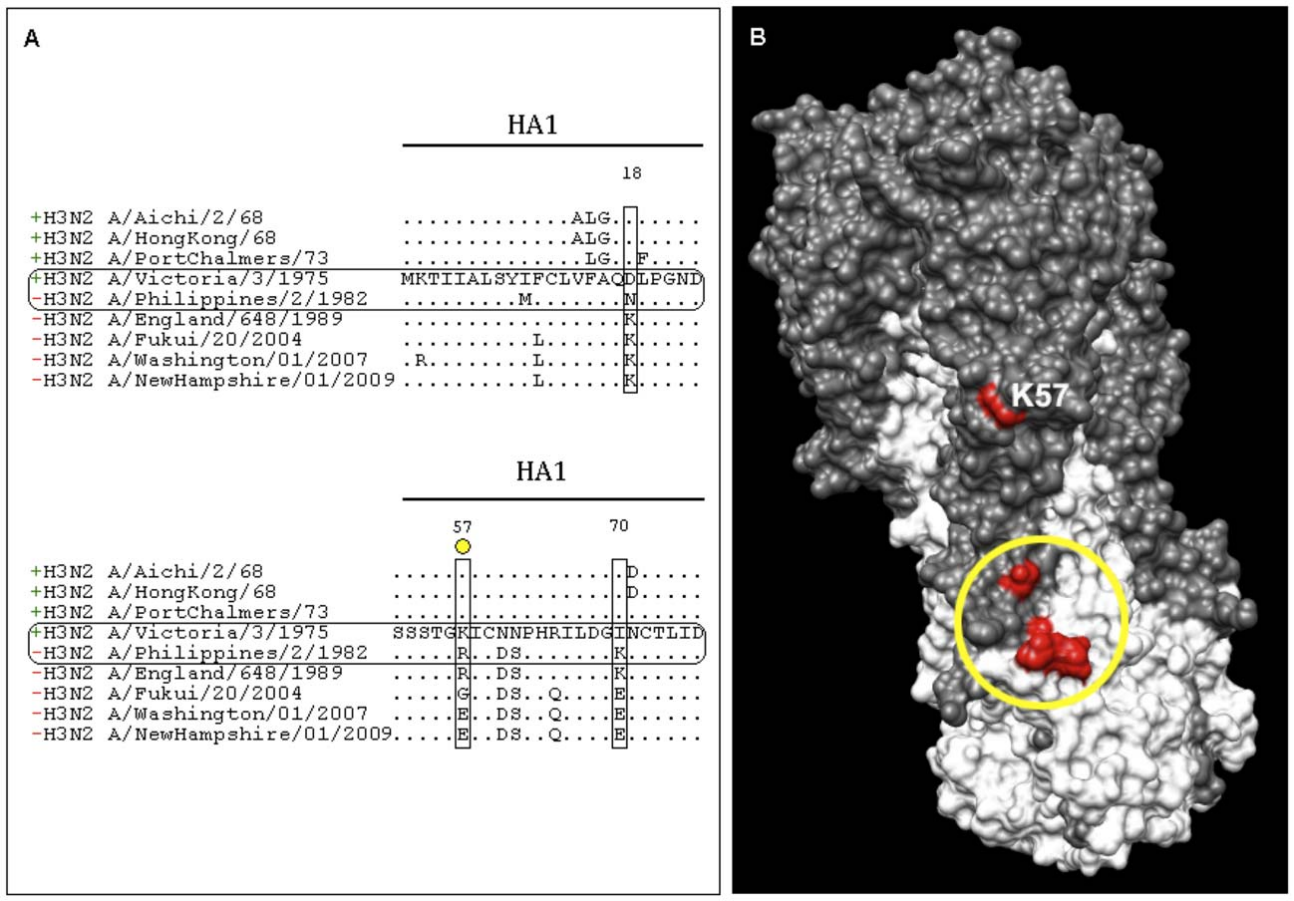
Sequence analysis of H3N2 HA1-hemagglutinins of viral isolates neutralized and not neutralized by PN-SIA28. (A) Aminoacidic sequences of H3N2 strains studied in the paper and in proximity to the region identified on H1N1 hemagglutinin were aligned. Differences among solvent exposed residues close to PN-SIA28 epitope core are boxed. The yellow circle on Lys57Ala mutant indicate 50–75% PN-SIA28 binding compared to wild-type HA. (B) (H3N2) A/Aichi/2/68 hemagglutinin trimer: grey areas indicate the HA1 domains whereas white areas indicate HA2 domains. Red regions indicate the residues able to inhibit PN-SIA28 binding to H3-HA. Yellow circle define the residues involved in the interaction of PN-SIA28 with both, H1 and H3 HAs.

A similar approach was followed also for the H3 subtype. Based on the results obtained for the H1N1 study, four H3 alanine mutants were initially generated on the A/Aichi/2/68 (H3N2) HA: His34Ala, Asn54Ala on HA1 and Ile363Ala and Asp364Ala on HA2 (corresponding to His25, His45, Ile361 and Asp362 in H1N1 sequencing numbering) (Figure 2, Figure 4 and Table S1). None of these alanine mutated HAs was bound by PN-SIA28. Furthermore, the Asn54His mutant (corresponding to position 45 in H1N1 numbering) on A/Aichi/2/1968 HA was also generated due to the presence of a Histidine in the corresponding position in H1N1 viruses. An increased binding of PN-SIA28 to this mutant was observed compared to wild type H3 HA. In order to deeper investigate on the absence of neutralizing activity of PN-SIA28 against most recent H3N2 strains, three more alanine-mutants (Asp18, Lys57 and Ile70) (see Table S1) were generated considering the differences in the stem region between H3N2 A/Victoria/3/1975 (the most recent neutralized isolate) and H3N2 A/Philippines/2/1982 (the earliest non neutralized strain). A 30% binding decrease was observed for Lys57Ala HA mutant, while the Asp18Ala and Ile70Ala did not have any effect on PN-SIA28 binding to H3N2 HA (Figure 5 and Table S1).

## Discussion

In the present study we describe the binding features and the neutralizing activity of a human monoclonal antibody named PN-SIA28 previously described as Fab fragment [19], [20]. IgG PN-SIA28 was tested against a large panel of influenza A viruses belonging to group 1 (H1N1, H2N2, H5N1, H9N2) and group 2 (H3N2, H7N2) subtypes showing a broader neutralizing activity compared to its monovalent molecule, possibly due to the bivalency feature of the IgG. Indeed, IgG PN-SIA28 showed a robust neutralizing activity (IC_50_ = 0.4–3.7 µg/ml) against all tested strains belonging to group 1, thus demonstrating that its epitope is broadly shared among group 1 viruses, including the highly divergent H9 subtype. More importantly, PN-SIA28 showed a potent neutralizing activity also against group 2 viruses demonstrating that its epitope is also present in the highly divergent H3N2 subtype. Indeed, IgG PN-SIA28 was able to potently neutralize H3N2 strains circulating from 1968 to 1975 (IC_50_ = 0.8–2.6 µg/ml), despite the important phylogenetic distance between these viruses and those belonging to group 1 (Figure 1). No neutralizing activity was observed against the dramatically divergent H7N2 isolate tested in this study, nor against the more recent H3N2 isolates.

The broad neutralizing activity of PN-SIA28 against highly divergent influenza viruses, suggests that the epitope recognized is extremely conserved. The lack of hemagglutination inhibitory activity and the competition with a mouse mAb (C179) directed against the stem region of HA, had already suggested that PN-SIA28 epitope is not localized on the globular head and that it is shared between HA1 and HA2 domains [19], [20]. Several approaches have been used in this study to better define the region recognized by PN-SIA28. The screening of a phagemidic random peptide library suggested the binding of PN-SIA28 to the stem region in the proximity of the viral membrane. Interestingly, the sequence analysis of A/PR/8/34 (H1N1) escape mutants, generated under PN-SIA28 selective pressure, identified two residues (Ile361 and Asp362) localized in close proximity to the residues identified with the peptide library approach. It is worth noting that none of these two specific mutations is present in any of the more than 6,000 sequences available in public influenza databases (http://www.ncbi.nlm.nih.gov/genomes/FLU/FLU.html), suggesting that the immune pressure on these residues is poor and that the region is functionally highly conserved in all subtypes (rate of non-conservative substitutions: 0.17% for residue 361, and 8.7% for residue 362). Based on these data, A/PR/8/34 (H1N1) HA mutants carrying an alanine substitution in position 361 (Ile361Ala) or in position 362 (Asp362Ala) were generated and the FACS analysis showed that PN-SIA28 lost most of its capability to bind the mutated HA, in concordance with the data obtained from the escape mutants. The residue in position 361 was also mutated in Valine (Ile361Val), an aminoacid largely present in most of the neutralized isolates (Figure 2), indeed confirming that the presence of this residue did not affect binding.

With the help of available HA crystal structures, a larger panel of HA-A/PR/8/34 (H1N1) alanine mutants was analogously generated and several residues (His25Ala, His45Ala on HA1; Thr358Ala, Met360Ala, Ile361Ala, Asp362Ala, Gly363Ala, Trp364Ala, Thr384Ala and Val395Ala on HA2), close to those identified with the escape mutants approach, influenced the interaction of PN-SIA28 with HA (Figure 2 and 3). The more restricted panel of alanine mutants (His25Ala, Asn45Ala, Ile361Ala and Asp362Ala) generated for A/Aichi/2/68 (H3N2) HA, confirmed the key role of some of the residues already identified as implicated in the interaction of PN-SIA28 with HA (H1) (Figure 4). Three more mutants were generated considering the differences among solvent exposed residues close to PN-SIA28 epitope-core on HA stem region between A/Victoria/3/1975 (the most recent neutralized isolate) and A/Philippines/2/1982 (the earliest non neutralized strain) isolates. The only mutant partially affecting PN-SIA28 binding was the one on Lys57, a residue present among all H3N2 neutralized strains and substituted by several different residues among H3N2 not neutralized strains. This could partially explaining the lack of neutralizing activity against most recent strains (Figure 5 and Table S1).

Overall, these results indicate that the region bound by PN-SIA28 encompasses residues on the HA stem, and that it is partially overlapping from the binding region of other human neutralizing antibodies with heterosubtipic neutralizing activity limited to influenza A group 1 viruses [25], [26], [27], [28], [29] or group 2 viruses [30], [31]. In addition, the region bound is also partially shared with FI6v3, an optimized human mAb able to cross-neutralize influenza A group 1 and group 2 viruses [32]. Importantly, both the heavy chains of PN-SIA28 and FI6v3 derive from rearrangement of the same VH gene germline (VH3-30). As already hypothesized for the VH1-69 subfamily, widely shared among human mAbs with heterosubtypic neutralizing activity limited to group 1 viruses, the VH3-30 gene structure may be important in conferring the broader heterosubtypic activity observed for PN-SIA28 and FI6v3. The future definition of HA/PN-SIA28 crystal structure and its comparison with HA/FI6v3 complex may help confirm the possible role of conserved VH3-30 residues in conferring such unusual neutralization properties.

Although the resolution of the crystal structure will be necessary for the fine definition of the PN-SIA28 epitope, several important considerations may already be made. Firstly, it was previously speculated that the presence of an additional glycosilation site (Asn54 corresponding to His45 present on group 1 viruses) on the HA1 portion of the stem region in H3N2 viruses is the reason for the lack of anti-H3N2 activity of other mAbs with heterosubtypic activity limited to group 1 subtypes [25], [26], [30]. The present study demonstrates that this extra glycosilation site on H3N2 does not preclude PN-SIA28 binding and neutralizing activity against viruses belonging to this subtype (Figure 2). The testing of HA alanine mutants for position His45 on H1N1 and Asn54 on H3N2, and the increased binding of PN-SIA28 to Asn54His mutant on H3N2, confirm that this key residue is particularly important for the interaction of PN-SIA28 with HA. However, other residues, including Lys57 and others not yet identified, certainly play an important role in the PN-SIA28 binding to HA, as shown by the lack of activity observed against the recent H3N2 isolates tested, as well as against the H7N2 isolate, despite a substantial identity in the studied region (Figure 2).

In conclusion, the molecule described in this paper is a singular human mAb featuring a broad heterosubtypic neutralizing activity encompassing group 1 and group 2 influenza A subtypes, and therefore recognizing a broad-range neutralizing human B epitope in the stem region of HA. Indeed, PN-SIA28 neutralizes influenza viruses belonging to all subtypes that have caused pandemics in humans, as well as other subtypes with pandemic potential.

The data presented may therefore be crucial not only for the improvement of classical passive anti-influenza prophylactic and therapeutic strategies [33], [34], but also for the correct understanding of mechanisms leading to *in vivo* protective anti-influenza immunity and therefore for the design of more effective vaccinal strategies. Indeed, several pharmacokinetic studies have shown that the extremely low IC_50_ featured by PN-SIA28 against most of the isolates tested can easily be reached in humans after systemic administration [35], [36]. Given the frequent correlation between a low IC_50_ value and *in vivo* efficacy observed for other anti-influenza mAbs [25], [26], [27], [28], [29], [30], [31], [37], [38], [39], PN-SIA28 could be a really promising compound to be used in passive immunization strategies.

More importantly, PN-SIA28 represents the molecular evidence that an extremely wide unusual neutralizing immunity although, uncommon, may be elicited during the course of a natural infection and, potentially, even after a new-generation vaccinal approach focused on its epitope [40]. As a consequence, PN-SIA28 could be crucial in the identification of promising HA-based vaccinal strategies aimed at the elicitation of a broadly heterosubtypic protective immunity similar to the one represented by this human mAb. As described for a few other mAbs [25], [26], [27], [28], [29], [30], [31], [32], [41], PN-SIA28 is mainly directed against the highly conserved HA2 domain whose use in novel anti-influenza vaccinal approaches is being widely investigated. However, PN-SIA28 demonstrates that not all antibodies directed against the stem region have the same characteristics, and that their neutralizing activity is modulated by the specifically recognized epitope. Indeed, the data presented in this paper show that in the design of such innovative approaches (i.e. the so-called “headless approach” [16]) it is important to identify also residues belonging to HA1 domain to be preserved to elicit the widest immunity. Several immunogens are usually obtained on the basis of *in silico* analysis, but their real protective potential can only be ascertained after time-consuming and expensive *in vivo* studies. Under this perspective, PN-SIA28 could therefore be an extremely important reagent to identify immunogens more likely to stimulate antibodies with a similar broad heterosubtypic neutralizing activity that will constitute the best candidates for further *in vivo* studies.

## Materials and Methods

### Monoclonal antibodies

The isolation of PN-SIA28 has been previously described [19], [20]. In brief, PN-SIA28 was isolated from a 55 year old patient with a negative clinical history of infection from influenza viruses during the past ten years, and with a detectable serum neutralizing titre (half maximal inhibitory dilution − ID_50_ > = 1∶20) against two reference strains belonging to two distinct HA-based phylogenetic groups: the H1N1 strain A/Puerto Rico/8/1934 for group 1 and the H3N2 strain A/Port Chalmers/1/1973 for group 2. It was then expressed in *E. coli* as Fab fragment to define its features.

To obtain the whole IgG described in this study, the BD BaculoGold System (BD Biosciences Pharmingen, San Diego, CA, USA) was used. Briefly, nucleotide sequences codifying heavy and light chains of PN-SIA28 Fab fragment were sub-cloned into the baculovirus expression vector pAc-κ-Fc (PROGEN Biotechnik GmbH, Heidelberg, Germany). Sf-9 insect cells (Invitrogen, Carlsbad, CA, USA) were co-transfected with the linearized baculovirus DNA (BD Biosciences Pharmingen, San Diego, CA, USA) and the pAc-κ-Fc/PN-SIA28. The obtained virus was employed at a molteplicity of infection (MOI) of 5 to infect 1×10^9^ H5 insect cells (Invitrogen, Carlsbad, CA, USA) in a final volume of 1 liter. After an incubation of 96 hours the culture media was collected, clarified by centrifugation and filtered with 0.2 µm filter (Millipore, Billerica, MA, USA). The media was loaded into a protein G column (Amersham Biosciences GE Healthcare, Zurich, Switzerland), the antibody was eluted with citric acid 0.1 M, pH 3, and immediately neutralized with Tris Base 1 M, pH 9. The solution containing the antibody was dialyzed against PBS and then concentrated using Amicon Ultra-15 Centrifugal Filter Devices (Millipore, Billerica, MA, USA). Antibody concentration was determined by SDS-PAGE gel and by spectrophotometric measurement at 280 nm.

An anti-influenza A antibody directed against the HA (H1N1 subtype), named RB62, and an anti-HCV E2 glycoprotein antibody, named e137, produced and purified with an identical procedure were used as controls in all experiments.

### Viruses and cells

The following reference strains were tested in the BLS3 laboratory of the Vita-Salute San Raffaele University: (H1N1) A/Puerto Rico/8/1934 (ATCC n. VR-1469); (H1N1) A/Wilson Smith/1933 (ATCC n. VR-1520); (H1N1) A/Malaya/302/1954 (ATCC n. VR-98); (H3N2) A/Hong Kong/8/1968 (ATCC n. VR-544); (H3N2) A/Aichi/2/1968 (ATCC n. VR-547); (H3N2) A/Victoria/3/1975 (ATCC n. VR-822); (H3N2) A/Port Chalmers/1/1973 (ATCC n. VR-810). The swine origin influenza virus (S-OIV) (H1N1v) A/Milan/UHSR1/2009 was previously isolated in our laboratory [19], [20]. A swine strain ((H1N1) A/swine/Parma/1/1997) was kindly provided by the Zooprophylactic Institute of Brescia, Italy. The following H2N2, H3N2, H5N1, H7N2 and H9N2 viruses were tested at the Centers for Disease Control and Prevention of Atlanta, Georgia, USA: (H2N2) A/Ann-Arbor/6/1960; (H3N2) A/Philippines/01/1982; (H3N2) A/England/648/1989; (H3N2) A/Fukui/20/2004; (H3N2) A/Washington/01/2007; (H3N2) A/NewHampshire/01/2009; (H5N1) A/Vietnam/1203/2004 clade 1; (H5N1) A/duck/Vietnam/NCVD98/2007 clade 2.3.4; (H7N2) A/New York/107/2003; (H9N2) LAIV A/chicken/Hong Kong/G9/1997.

All viruses, excluding the A/swine/Parma/1/1997, were cultured on MDCK (Madin-Darby Canine Kidney) (ATCC CCL-34) cells propagated in Modified Eagle Medium (MEM) (Gibco Invitrogen, Carlsbad, CA, USA) supplemented with 10% inactivated fetal bovine serum (Gibco Invitrogen, Carlsbad, CA, USA), 50 µg/ml of penicillin (Gibco Invitrogen, Carlsbad, CA, USA), 100 µg/ml of streptomycin (Gibco Invitrogen, Carlsbad, CA, USA). The A/swine/Parma/1/97 isolate was analogously grown on NSK (Newborn Swine Kidney) cells, kindly provided by the Zooprophylactic Institute of Brescia, Italy. At 80% confluence, cells in MEM supplemented with 2 µg/ml serum-free TPCK-trypsin (Roche Applied Science), were infected with each strain at a MOI of 0.001. After 1 hour of infection, cells were washed with PBS (Phosphate buffered saline); MEM supplemented with 2 µg/ml trypsin was then added and cells were incubated at 37°C in 5% CO_2_ atmosphere. Cells were observed daily to monitor the cytopathic effect and, usually after 72–96 hours, the supernatant was collected, centrifuged at 1000 rcf for 10 minutes to eliminate cells debris and filtered with 0.22 µm filters (Millipore, Billerica, MA, USA). The supernatant was then aliquoted and stored at −80°C as cell-free virus stock.

### Virus neutralization assays

#### Fluorescence inhibition assay

Each viral isolate was titrated by the limiting dilution method and the viral titer calculated by the Reed-Muench formula. Neutralizing assays were carried out in 96 wells plate using MDCK cells (4×104cells/well). Serial dilutions, 30 µg/ml-0.03 µg/ml, of IgG PN-SIA28 were preincubated for 1 hour at 37°C with 100 TCID50 of each H1N1 or H3N2 virus. Following incubation, 100 µl of the mix antibody-virus were added to the cells and incubated for another hour at 37°C in 5% CO2. At the end of this incubation, cells were washed with PBS and 100 µl of MEM TPCK-Trypsin (2 µg/ml) were added in each well. Cells were incubated for 7 hours at 37°C in 5% CO2 and then washed with PBS, fixed and permeabilized with ice-cold ethanol. Cells were incubated with anti-influenza A mouse antibody (Argene, Shirley, NY, USA) for 30 minutes at 37°C in a humid chamber. The cells were then washed and incubated for 30 minutes at 37°C in a dark humid chamber with a FITC-conjugated secondary antibody (Argene, Shirley, NY, USA). Nuclei staining was obtained with Hoechst 33342 (Sigma Aldrich). An infection control without antibody was included, as well as a negative control with an anti-HCV/E2 antibody (e137). Each neutralization assay was performed in triplicate and repeated in two different sessions.

The neutralization activity for each antibody concentration was expressed as the percentage reduction of fluorescent nuclei compared with the nuclei count in the infection control. Nuclei counting was performed by using the GE Healthcare's IN Cell Analyzer 1000, an automated epifluorescence based microscope system. The neutralization curves were then fit by non-linear regression with the GraphPad Prism software, allowing IC_50_ calculation.

#### Colorimetric assay

Each viral isolate was titrated to establish working dilution that produces 15–30 foci forming units per well in 96 tissue culture plates. Neutralizing assays were carried out in 96 wells plate using MDCK/SIAT-1 cells. Serial dilutions, 30 µg/ml-0.37 µg/ml, of IgG PN-SIA28 were preincubated for 1 hour at 37°C with the subset of viruses. Following this incubation, 100 µl of the antibody-virus mix was added to the cells and incubated for another hour at 37°C in 5% CO_2_. At the end of this incubation, the cells were washed twice in PBS and 100 µl of virus growth media containing 2 µg/ml of TPCK treated trypsin was added. Cells were incubated for 12–16 hours at 37°C in 5% CO_2_ and then washed with PBS, fixed and permeabilized with ice cold methanol/acetic acid (95∶5) for 30 min at −20°C. Cells were incubated with anti-NP antibodies (Millipore, Billerica, MA, USA) for 30 minutes at 37°. The cells were then washed and incubated for 30 additional minutes at 37°C with a mouse HRP-conjugated secondary antibody. True Blue chromogenic substrate (KPL) was used to count the number of foci.

#### Plaque reduction assay

Each viral isolate was titrated by the limiting dilution method and the viral titre calculated by the Reed-Muench formula. The viral titre was also calculated as plaque forming units (PFU) on six-well flat-bottomed plates (Corning, Corning, NY, USA). Neutralizing assays were carried out in 6 wells plates using MDCK cells (5×10^5^ cells/well). Two dilutions, 1–0.1 µg/ml, of IgG PN-SIA28 were preincubated for 1 hour at 37°C with 100 TCID_50_ each of H1N1 or H3N2 virus. Following this incubation, 1 ml each of virus-antibody mix was added on MDCK monolayer and the plate was incubated 1 hour at 37°C in 5% CO_2_. After this incubation, the medium was removed and the monolayer washed twice with PBS. Two ml of MEM-agarose 0.8% supplemented with penicillin (50 µg/ml) (Gibco Invitrogen, Carlsbad, CA, USA), streptomycin (100 µg/ml) (Gibco Invitrogen, Carlsbad, CA, USA), L-glutamine (2 mM) (Gibco Invitrogen, Carlsbad, CA, USA) and trypsin (2 µg/ml) (Roche Applied Sciences) were gently added to each well and the plates were incubated 48 hours at 37°C in 5% CO2. After this incubation the agarose medium was removed from each well and 1 ml of 70% methanol-crystal violet 1% (w/v) was added to each well at room temperature. Finally, the wells were washed with tap water and dried. An infection control without antibody was added as well as a negative control with anti-HCV/E2 e137 mAb. The neutralization was determined counting the PFU reduction in presence of antibodies in comparison with the infection control.

### Peptide panning

A phagemidic 12-mer peptide library (Ph.D.12™ Phage Display Peptide Library, New England Biolabs Inc., Boston, MA, USA) was screened against PN-SIA 28 Fab fragment. Briefly, 300 ng of PN-SIA 28 were coated on four wells (96-wells plate, COSTAR), and the peptide library was amplified transforming electrocompetent *E. coli* XL1 Blue. 70 µl of the obtained phage preparation was incubated with PN-SIA 28 for 1 hour at 37°C and subsequently the unbound phages were removed by washing with PBS 1X-Tween20 using progressive Tween20 concentration starting from 0.1% to 0.5%. The phage that bound PN-SIA 28 was then eluted using low pH and, once neutralized, used to infect *E. coli* XL1 Blue for a subsequent ELISA screening on 100 ng/well coated PN-SIA 28. Positive clones were then sequenced using the kit-provided primers (New England Biolabs Inc., Boston, MA, USA).

Positive clones were then sequenced. An *in silico* analysis was performed, using Mimox and Pepitope servers, comparing the selected peptides to the available crystal structure of A/PR/8/34 and A/South Carolina/1918 haemagglutinins (1RU7.pdb and 1RD8.pdb).

### Selection and characterization of antibody escape mutants under selective pressure of antibody PN-SIA28

The experiment was performed on 90% confluent MDCK cells growing in T25 flasks (Nunc, Rochester, NY, USA) in MEM supplemented with 10% FBS. PN-SIA28 as well as e137 mock control, were diluted in 1.5 mL MEM supplemented with TPCK trypsin (2 µg/ml) to obtain final concentrations of 2 µg/ml, 10 µg/ml and 20 µg/ml of antibody. 100 TCID_50_ of A/PR/8/34 were prepared in 1.5 mL of the same medium. The two solutions, containing antibodies and virus, were mixed in order to obtain a final concentration of 1 µg/ml, 5 µg/ml and 10 µg/ml for the mAbs in a final volume of 3 mL. The mixes were then incubated 1 h at 37°C. An infection positive control (virus without PN-SIA28) was included as well as non-infected cells. After two washes with sterile PBS 1× (Gibco Invitrogen, Carlsbad, CA, USA), 1 ml of each neutralization mix was added to the flasks containing MDCK cells and the infection was performed for 1 h at 34°C in 5% CO_2_. After absorption, the medium was removed and the monolayer washed twice with sterile PBS. 3 mL of MEM supplemented with trypsin (2 µg/ml) was added to the infection positive control and to non-infected cells. Antibody PN-SIA28 and e137 were added to the previously treated infected cells, maintaining the concentrations used during the infection step. Cells were incubated at 34°C in 5% CO_2_ and regularly checked for 48 hours for the presence of cytopathic effect (CPE) by comparing the positive infection control to the treated infected cells. The supernatant was then centrifuged (2000 rcf for 10 minutes), collected and stored at −80°C. All the viral stocks were titrated and used to infect new cell preparations, increasing, when it was possible, the concentration of PN-SIA28. After ten passages, the cells of the infection positive control and mock control were compared with infected cells under selective pressure of PN-SIA28. Once a strong cytopathic effect was evident in the positive and mock controls, the presence or absence of CPE in the PN-SIA28 treated infected cells was evaluated. All the supernatants were collected, centrifuged, stored and used to perform a full length DNA sequence of influenza genomic fragment 4 coding for viral HA.

### HA cloning and mutagenesis

A/PR/8/34 (H1N1) HA was amplified as previously described [19], [20] using the following PCR-oligonucleotides: APR834_s: 5′-CACCATGAAGGCAAACCTACTGGTCCTGTTATGTG-3′; APR834_as: 5′-TCAGATGCATATTCTGCACTGCAAAGATCCATTAGA-3′. A/Aichi/2/1968 (H3N2) HA was amplified using the following PCR-oligonucleotides: Aichi/2/68H3N2f: 5′-CACCATGAAGACCATCATTGCTTTG-3′; Aichi/2/68H3N2r: 5′-TCAAATGCAAATGTTGCACCTAATG-3′. The PCR products were cloned into the pcDNA 3.1D/V5-His-TOPO vector (Invitrogen, Carlsbad, CA, USA). Subsequently, alanine mutants for H1N1 and H3N2 HA were generated using Gene Tailor Site-Directed Mutagenesis System (Invitrogen, Carlsbad, CA, USA). A total of 20 HA mutants were generated for H1N1 and 7 for H3N2 (Table S1).

### Cytofluorimetric binding assays

The binding activity of PN-SIA28 was assayed using full-length wild type and mutants HAs cloned as described above. In brief, 1×10^6^ human epithelial kidney (HEK) 293T cells (ATCC CRL-1573) were transfected in 6 wells plate (Corning, Corning, NY, USA) with 4 µg of pcDNA 3.1D/V5-His-TOPO vector containing the HA nucleotide sequences. After centrifugation and fixation with paraformaldehyde 4%, the transfected cells were incubated for 30 minutes at room temperature with PN-SIA28 or a conformational control for H1N1 (RB62) or a control for H3N2 (12D1, kindly provided by P.Palese) at 1 µg/ml and 10 µg/ml. Additionally, the isotype control, e137 (1 and 10 µg/ml) was introduced as well as untransfected cells and a mouse anti-H1 subtype monoclonal antibody directed against a linear epitope (anti-influenza A hemagglutinin [12D1102] GeneTex Inc., Irvine, CA, USA) to evaluate the transfection efficiency for each HA. The cells were then washed and incubated for 30 minutes at room temperature with FITC-conjugated anti-human (Sigma Aldrich) or anti-mouse (Argene, Shirley, NY, USA) monoclonal antibodies. Afterwards, the cells were washed and analysed by FACS. The FACS data were analyzed using the software Weasel w 2.5 (Waler+Eliza Hall, Institute of Medical Research, Parkville Victoria, Australia). The binding of PN-SIA28 to the different HA-mutants was expressed as a binding percentage compared to wild-type.

### Software

For sequences analysis the following software packages were used: SeqScape (Applied Biosystems), ClustalX (Toby Gibson), Bio Edit (Tom Hall, Ibis Therapeutics), and Treeview (GubuSoft). For molecular visualization and rendering UCSF Chimera package from the Resource for Biocomputing Visualization and Informatics at University of California, RasMol (Roger Sayle), Jmol (Jmol: an open-source Java viewer for chemical structures in 3D. http://www.jmol.org/), Cn3D (United States National Library of Medicine, NLM), Pepitope server (Pepitope: epitope mapping from affinity-selected peptides. Bioinformatics 2007 23(23):3244-3246.), Mimox (*BMC Bioinformatics* 2006, **7**:451 doi:10.1186/1471-2105-7-451) were used. Finally for data analysis and graphical editing GraphPad Prism was used.

## Supporting Information

Figure S1 **Neutralization assays against viruses from group 1 subtypes.** Dose–response curve fit nonlinear regression is reported for PN-SIA28 against H1N1, H5N1, H2N2 and H9N2 strains studied in this paper. Data from two different experiments for each strain are reported. Each point was performed in triplicate.
(TIF)

Figure S2 **Neutralization assays against H3N2 viruses.** Dose–response curve fit nonlinear regression is reported only the H3N2 viruses neutralized by PN-SIA28. Data from two different experiments for each strain are reported. Each point was performed in triplicate.
(TIF)

Figure S3 **Unrooted tree of HA1 region of H3 hemagglutinins.** The different isolates belong to different historical periods spanning 1968 to 2009. The three different clusters characterizing the H3N2 pandemic are evidenced, with each cluster including isolates belonging to a distinct time period of the pandemic. Green ‘+’ or red ‘−’ indicate PN-SIA28 positive and negative neutralizing activity, respectively.
(TIF)

Table S1 HA amino acidic residues mutated in H1 and H3.
(DOC)
