# Supplementary figures and images for: A Human Monoclonal Antibody with Neutralizing Activity against Highly Divergent Influenza Subtypes
Source: PLoS One. 2011 Dec 5;6(12):e28001. doi: 10.1371/journal.pone.0028001 (PMC3230632; doi:10.1371/journal.pone.0028001)

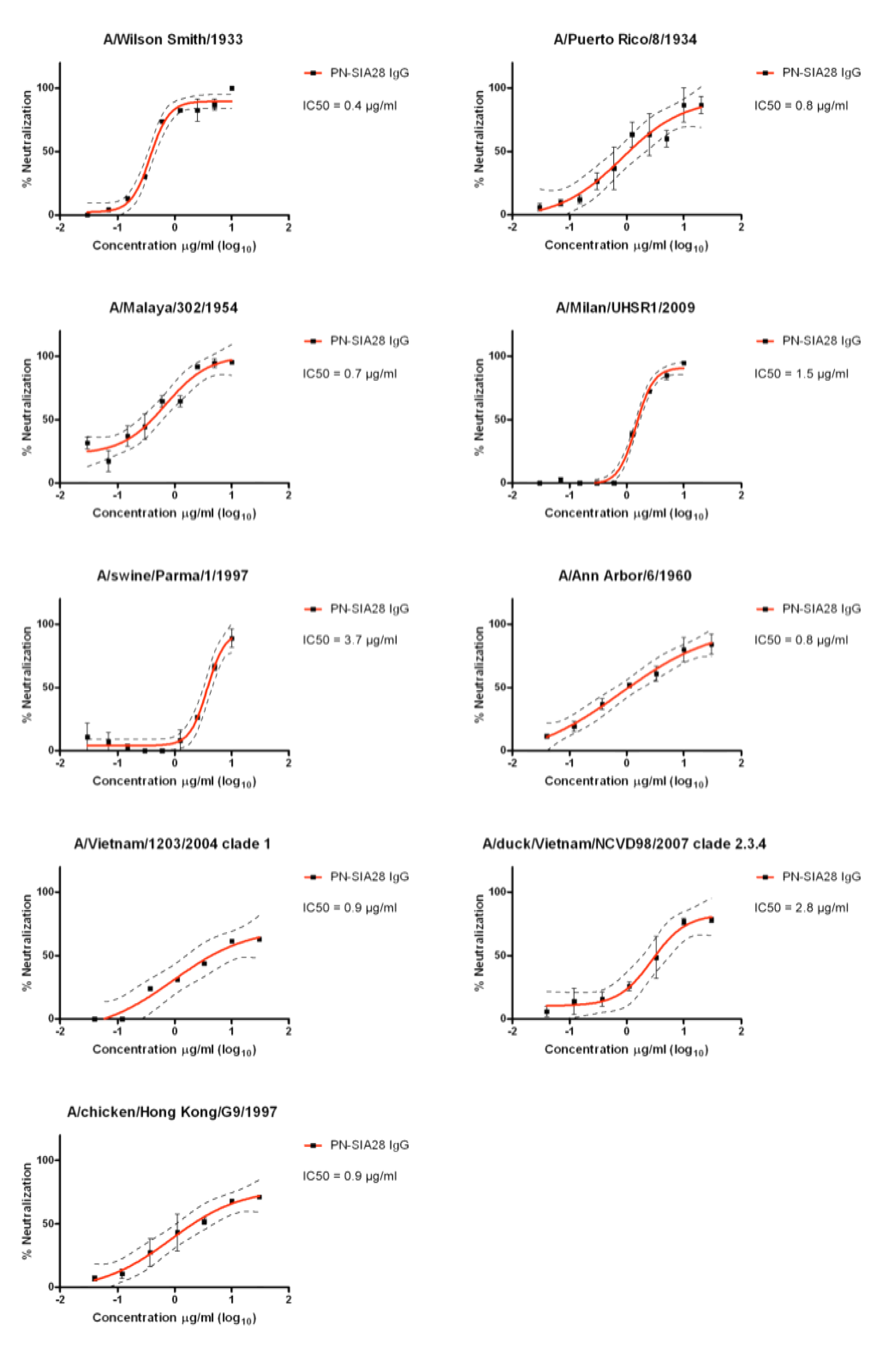

Supplement: Figure S1 — Neutralization assays against viruses from group 1 subtypes. Dose–response curve fit nonlinear regression is reported for PN-SIA28 against H1N1, H5N1, H2N2 and H9N2 strains studied in this paper. Data from two different experiments for each strain are reported. Each point was performed in triplicate. (TIF) [file pone.0028001.s001.tif]

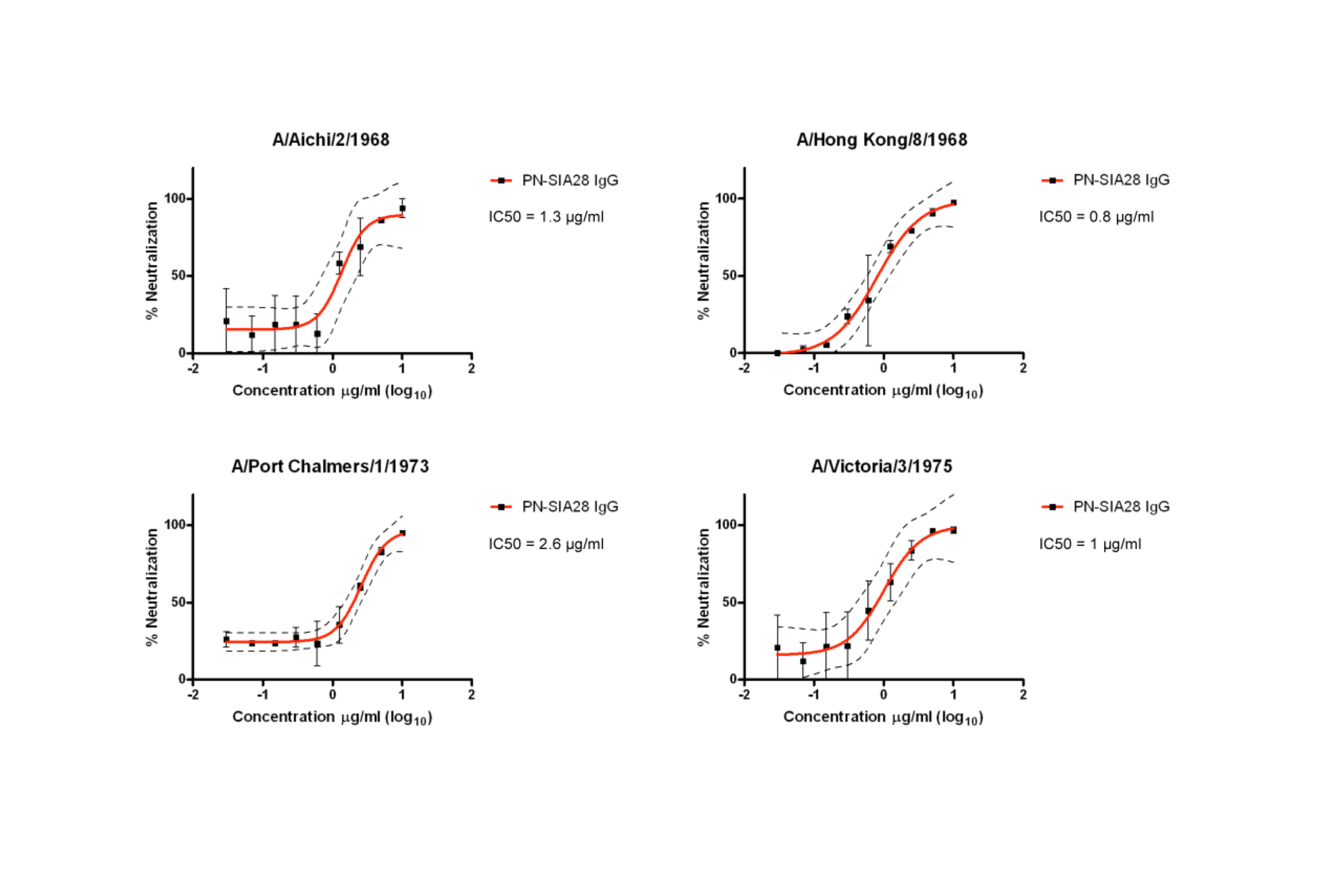

Supplement: Figure S2 — Neutralization assays against H3N2 viruses. Dose–response curve fit nonlinear regression is reported only the H3N2 viruses neutralized by PN-SIA28. Data from two different experiments for each strain are reported. Each point was performed in triplicate. (TIF) [file pone.0028001.s002.tif]

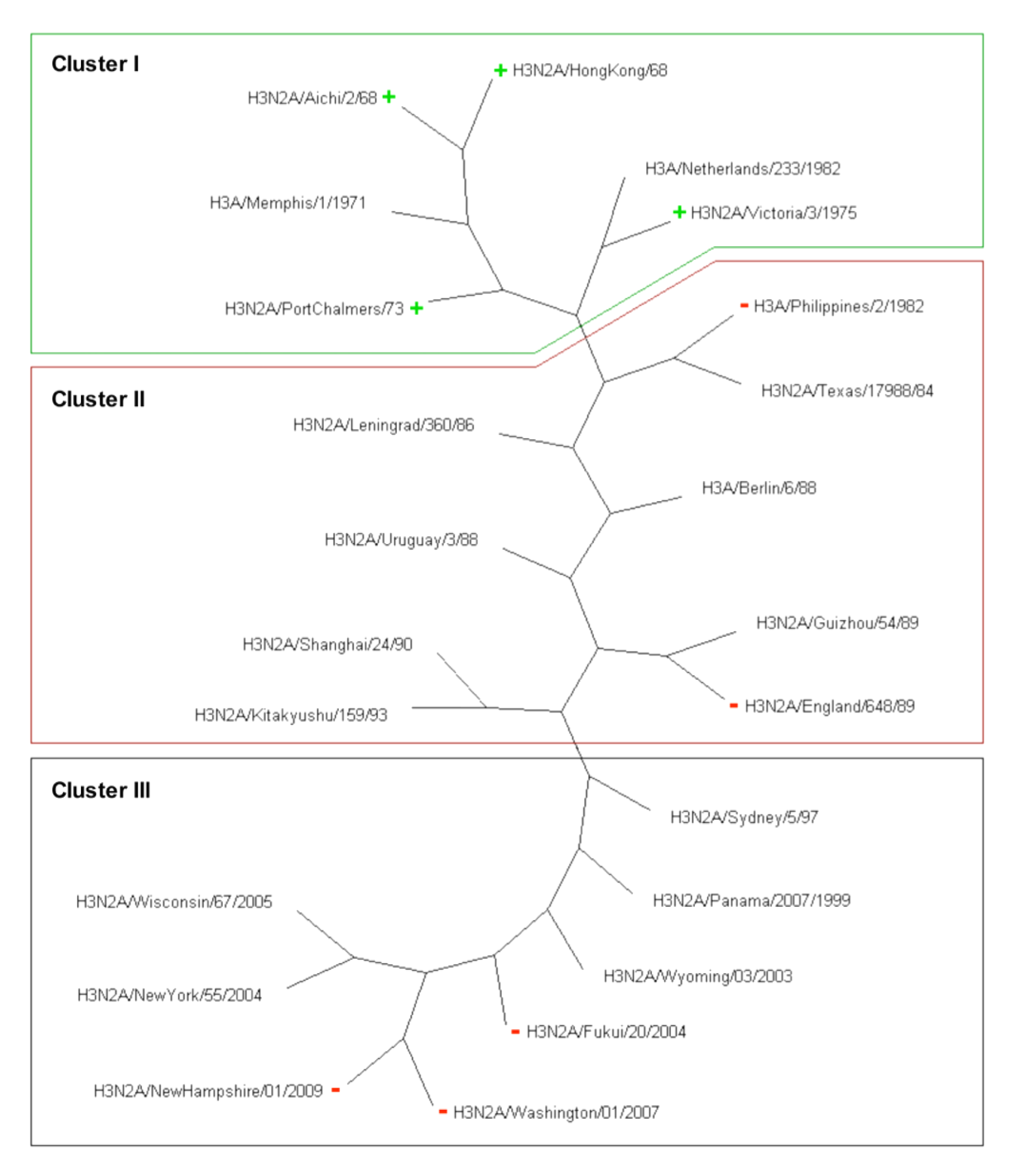

Supplement: Figure S3 — Unrooted tree of HA1 region of H3 hemagglutinins. The different isolates belong to different historical periods spanning 1968 to 2009. The three different clusters characterizing the H3N2 pandemic are evidenced, with each cluster including isolates belonging to a distinct time period of the pandemic. Green ‘+’ or red ‘−’ indicate PN-SIA28 positive and negative neutralizing activity, respectively. (TIF) [file pone.0028001.s003.tif]
