# Supplementary material for: A Human Monoclonal Antibody with Neutralizing Activity against Highly Divergent Influenza Subtypes
Source: PLoS One. 2011 Dec 5;6(12):e28001. doi: 10.1371/journal.pone.0028001 (PMC3230632; doi:10.1371/journal.pone.0028001)
Supplement: Table S1 — HA amino acidic residues mutated in H1 and H3. (DOC) [file pone.0028001.s004.doc]

**Table S1. HA amino acidic residues mutated in H1 and H3.**

| **HA-subtype** | **HA-subunit** | **Residue** |
| --- | --- | --- |
| H1N1 | HA1 | His25 |
|  |  | His45 |
|  |  | Thr315 |
|  |  | Asn336 |
|  |  | Ile337 |
|  |  | Pro338 |
|  | HA2 | Trp357 |
|  |  | Thr358 |
|  |  | Gly359 |
|  |  | Met360 |
|  |  | Ile361^a^ |
|  |  | Asp362 |
|  |  | Gly363 |
|  |  | Trp364 |
|  |  | Thr384 |
|  |  | Ile388 |
|  |  | Thr392 |
|  |  | Val395 |
|  |  | Asn396 |
|  |  | Glu400 |
| H3N2 | HA1 | Asp18^b^ |
|  |  | His25^c^ |
|  |  | Asn45^d^ |
|  |  | Lys57^e^ |
|  |  | Ile70^f^ |
|  | HA2 | Ile361^g^ |
|  |  | Asp362^h^ |

^a^For residue Ile361 two HA mutants were generated: Ile361Ala and Ile361Val. See main text for details ^b^Asp18 not present on A/PR/8/34 HA aminoacidic sequence

^c^His34 according to coding sequence numbering of (H3N2) A/Aichi/2/68 (GenBank accession number P03437)

^d^Asn54 on A/Aichi/2/68. For this residue two HA mutants were generated: Asn45Ala and Asn45His. See main text for details

^e^Lys66 according to coding sequence numbering of (H3N2) A/Aichi/2/68 (GenBank accession number P03437)

^f^Ile78 according to coding sequence numbering of (H3N2) A/Aichi/2/68 (GenBank accession number P03437)

^g^Ile363 on A/Aichi/2/68

^h^Asp364 on A/Aichi/2/68
